# Technology-based functional assessment in early childhood intervention: a pilot study

**DOI:** 10.1186/s40814-018-0260-1

**Published:** 2018-03-21

**Authors:** Mary A. Khetani, Beth M. McManus, Kristen Arestad, Zachary Richardson, Renee Charlifue-Smith, Cordelia Rosenberg, Briana Rigau

**Affiliations:** 1 Departments of Occupational Therapy and Disability and Human Development, University of Illinois at Chicago (UIC), 1919 West Taylor Street, Room 316A, Chicago, IL 60612-7250 USA; 2 Department of Health Systems, Management and Policy, University of Colorado Denver, Denver, CO USA; 3 Children’s Hospital Colorado, Colorado Springs, CO USA; 4 JFK Partners, University of Colorado School of Medicine, Aurora, CO USA; 5 Children’s Participation in Environment Research Lab (CPERL), University of Illinois at Chicago, Chicago, IL USA

**Keywords:** Technology assessment, Patient-reported outcome measures, Early intervention, Social participation, Performance, Feasibility

## Abstract

**Background:**

Electronic patient-reported outcomes (e-PROs) may provide valid and feasible options for obtaining family input on their child’s functioning for care planning and outcome monitoring, but they have not been adopted into early intervention (EI). The purpose of this pilot study was to evaluate the feasibility of implementing technology-based functional assessment into EI practice and to examine child, family, service, and environmental correlates of caregiver-reported child functioning in the home.

**Methods:**

In a cross-sectional design, eight individual EI providers participated in a 90-min technology-based functional assessment training to recruit participants and a 60-min semi-structured focus group post data collection. Participants completed the Young Children’s Participation and Environment Measure (YC-PEM) home section online and Pediatric Evaluation of Disability Inventory Computer Adaptive Test (PEDI-CAT) via iPad. Participants’ EI service use data were obtained from administrative records.

**Results:**

A total of 37 caregivers of children between 6 and 35 months old (mean age = 19.4, SD = 7.7) enrolled, a rate of 44% (37/84) in 2.5 months. Providers suggested expanding staff training, gathering data during scheduled evaluations, and providing caregivers and providers with access to assessment summaries. Caregivers wanted their child’s participation to change in 56% of home activities. Lower caregiver education and higher EI intensity were related to less child involvement in home activities.

**Conclusions:**

Implementing technology-based functional assessment is feasible with modifications, and these data can be useful for highlighting child, family, and EI service correlates of caregiver-reported child functioning that merit further study. Feasibility results informed protocol modifications related to EI provider training, timing of data collection, and management of EI service use data extraction, as preparation for a subsequent scale-up study that is underway.

## Background

According to Part C of the Individuals with Disabilities Education Act (IDEA), infants and toddlers with developmental delays and disabilities qualify for early intervention (EI) services in the USA [1]. EI care includes developmental and therapeutic (e.g., physical therapy) care to infants and toddlers with developmental delays and disabilities. Although EI services range in type and intensity [2], EI is a common source of rehabilitation for eligible families. The per child allocation in EI has decreased from $1979 in 1999 to $1258 in 2000 [3], resulting in greater restrictions on service eligibility [4, 5] and increased burden on EI providers to ensure optimal outcomes with fewer resources. The Office of Special Education (OSEP) requires states to report on child outcomes for accountability and quality improvement [6]. These outcomes address the child’s integration of developmental skills to function [6].

EI teams collect multiple sources of information to complete a child outcomes summary (COS) process for OSEP reporting [5]. Standardized developmental assessments are used to determine a child’s developmental level and EI eligibility. IDEA mandates states administer multidisciplinary (i.e., at least two different disciplines) and comprehensive (i.e., addresses the five neurodevelopmental domains) developmental evaluations. However, in practice, EI eligibility evaluation results primarily reflect a child’s capacities to perform discrete functional tasks (e.g., completing puzzle, walking steps). Although developmental information is needed to determine EI eligibility, this information is inadequate for evaluating the child’s functional status when planning EI care and monitoring outcomes.

Therefore, IDEA encourages EI providers to gather family input about child functioning for the COS process. Family input can be gathered through EI team observation of the child and/or caregiver interview. Observational assessment captures provider perspectives on the child’s current functioning in a subset of activity contexts, whereas caregiver interview yields family input about current and desired child functioning across a broad range of activity contexts [7–9]. While caregiver interviews allow EI providers to develop a more comprehensive functional profile of the child as well as capture family priorities for change, these interviews require two providers and last up to 1.5 h. Therefore, some families opt out of the caregiver interview, and when they do, family priorities are not available to the EI care team when developing an individualized family service plan to direct EI care and monitor meaningful EI outcomes [8].

Electronic patient-reported outcomes (e-PROs) may provide a valid and feasible alternative for obtaining family input on a child’s functioning to support care planning, outcomes monitoring, and research in EI [10, 11]. Technology-based functional assessment using e-PROs may maximize EI provider reach because families with internet access will experience portability, tailoring, privacy, and autonomy when providing input on their child’s functioning [12]. Yet, to date, e-PROs have not been adopted into EI.

There are two e-PROs that may help to resolve content and feasibility problems when obtaining family input for EI COS processes, as well as enable testing of links between EI service use and functional outcomes for advancing patient-centered outcomes research in EI. The Pediatric Evaluation of Disability Inventory Computer Adaptive Test (PEDI-CAT) is an e-PRO that enables caregiver assessment of the child’s functioning according to his or her ability to perform discrete tasks, whereas the Participation and Environment Measure (PEM) assessments are e-PROs that capture a child’s functioning according to caregiver satisfaction and perspectives of their child’s participation across activities and environments.

Both of these e-PROs facilitate family engagement by providing valuable information about family perspectives and priorities when planning and monitoring EI care. The PEDI-CAT and PEM e-PROs are also endorsed for patient-centered outcomes research in pediatric rehabilitation. PEDI-CAT is recommended for rehabilitation clinical trials involving young children with movement disorders [13, 14], and PEM questionnaires are recommended by NIH/NINDS as common data elements for intervention studies involving children with cerebral palsy [15]. In fact, a recent study employing PEDI-CAT and PEM e-PRO data showed the influence of child and family characteristics on home participation for children 0–5 years old could be explained, largely, by caregiver perceptions of environmental support [16]. The child’s environment, as measured by PEM, explained over 40% of the variance in the child’s level of involvement in home-based activities, which is more than prior studies measuring children’s participation [17]. However, these previous studies’ findings are not specific to young children with developmental delays and disabilities nor do they adjust for variability in EI service use. Hence, these findings warrant replication with an EI population while accounting for EI service use. These types of analyses can help to build clinically relevant knowledge about the adequacy of EI services on functional outcomes from the family perspective.

To our knowledge, this is the first study to pilot the PEDI-CAT and PEM e-PROs with an EI population to examine their feasibility and value. The primary aims of this pilot study were twofold: (1) to evaluate the feasibility of introducing e-PROs for technology-based functional assessment during routine EI care visits so that the need for protocol modifications could be determined (aim 1) and (2) to identify associations between EI service use and parent-reported outcomes of functioning that merit further investigation in a subsequent scale-up study (aim 2). Study results will contribute clinically relevant knowledge about the potential for e-PRO use for EI care planning and outcomes monitoring.

## Methods

### Design

This pilot study employed an observational, cross-sectional design.

### Setting

This study was conducted in partnership with an early intervention (EI) program located in the Denver Metro area of Colorado in the USA.

### Participants

Participants met four inclusion criteria: (1) primary caregiver of a child receiving EI services; (2) 18 years or older; (3) able to read, write, and speak in English; and (4) had a child enrolled in EI for 3 or more months.

### Data collection

Multi-institutional ethics approval was obtained. Eight EI providers (i.e., physical therapists, occupational therapists, speech therapists, and early childhood developmental specialists) completed a 90-min on-site training regarding subject recruitment and technology-based data collection prior to participant recruitment.

EI staff with active caseloads completed a 90-min on-site training prior to data collection. Training included project overview, feedback on recruitment materials, and orientation to e-PRO content and administration via iPad. EI providers issued eligible participants informational flyers during EI service visits. Participants who expressed interest consented and enrolled in the study online via personal computer or via iPad issued by the EI provider during the service visit. Eligible participants created user accounts and provided informed consent to enroll in a pilot study to test the feasibility of introducing e-PRO data collection and possible associations between EI service use and parent-reported outcomes of functioning that warrant further investigation in a subsequent larger study. The success of feasibility was determined as e-PRO enrollment and completion rates of 50% or higher based on family assessment completion rates within usual care, as well as provider perspectives on their ability to screen and enroll families during routine EI service visits.

Following informed consent, participants completed demographic and YC-PEM questionnaires online, followed by PEDI-CAT completion via iPad. HIPAA authorization was obtained for agency release of service use data from the EI program’s administrative database to examine the association between service use and e-PRO scores. Participants were mailed $40.00 gift cards.

During data collection, the EI staff lead issued weekly newsletters that were developed by the research staff. The EI staff lead also participated in bi-monthly phone calls with research staff to monitor study enrollment and completion rates and relay EI provider questions about resources and management to research staff as they arose during recruitment and data collection. Research staff conducted a 60-min semi-structured focus group with four EI staff following data collection to obtain provider perspectives on resources and management needed to conduct the protocol (see Appendix for interview guide).

### Measures

The primary measured outcome was feasibility, as defined by provider perspectives and ability to screen and enroll eligible families during scheduled EI visits, as well as e-PRO completion rates by families. Secondary outcome measures were two e-PROs of child functioning as clinically important endpoints for EI outcomes monitoring.

### Pediatric Evaluation of Disability Inventory-Computer Adaptive Test (PEDI-CAT)

PEDI-CAT [10] affords for caregiver assessment of functional task performance for children birth to 20 years old. Caregivers are asked about their child’s performance in specific tasks using a 5-point scale, from “unable” to “easy to do.” In this study, normative scores were generated for three out of four assessment domains: daily activities (68 items), mobility (97 items), and social/cognitive (60 items) [17]. PEDI-CAT domains have excellent test-retest reliability [18].

### Young Children’s Participation and Environment Measure (YC-PEM)

YC-PEM captures caregiver perspectives of their child’s participation in home, daycare/preschool, and community activities and environmental influences on participation. Participants completed the YC-PEM home section. Caregivers evaluated their child’s participation in 13 home-based activity types. For each type, caregivers reported on (1) participation frequency (8-point scale, from never [0] to once or more each day [7]); (2) their child’s level of involvement (5-point scale, from not very involved [1] to very involved [5]); and (3) their desire for change in their child’s participation (yes, no). Then, caregivers evaluated the impact of 13 features and resources in the home environment on participation (3-point scale, from no impact/usually helps/usually yes [3] to usually makes harder/usually no [1]).

The four YC-PEM home scales have internal consistencies ranging from good to excellent and adequate test-retest reliability [19]. Reliability estimates were also adequate for data obtained in this study (*α* = 0.70–0.77).

### Service records

Service use data were acquired, following caregiver consent, from the EI program’s administrative database and included information on total EI hours and total EI duration in months. Service intensity was derived for each case according to EI hours per month.

### Sample size

Target sample size was *n* = 42, or 50% of the total active enrollment at the EI program during the data collection period. For aim 1, this target sample size was deemed to be sufficient to examine how feasible the e-PRO assessment option is relative to the standard interview option that is completed by approximately 50% of families in the EI program. Additionally, the EI staff lead approved the target sample size upon considering provider ability to screen and enroll families during routine EI service visits. For aim 2, this target sample size would allow us to explore and generate hypotheses about EI service use and functional outcomes by replicating and expanding on prior models of children’s participation that employ a minimum of five independent variables (five cases per variable) [16, 17, 20].

### Data analysis

For aim 1 on feasibility outcomes, descriptive analyses were used to estimate feasibility of technology-based family assessment of children’s functioning. Enrollment rates and reasons for non-participation were reported as proportions based on the number of EI-enrolled families meeting inclusion criteria during the enrollment period (November 2015 to January 2016). Completion rates were reported as proportions based on the number of actively enrolled families in the current study. Focus group data were audio-recorded. Three research staff independently reviewed the recording to generate a written summary of recurrent EI provider suggestions for protocol improvement. Written summaries were then compared to determine that there were no discrepancies to address through discussion and consensus. Hence, the content across summaries were merged into a single written summary.

For aim 2 on functional outcomes, EI service use data were merged with demographic and e-PRO data and then imported into STATA 13.0. Caregiver and child demographic and service use characteristics were summarized using proportions and mean (standard deviation [SD]) as well as median (Q1, Q3) scores depending on sample distribution. Three cases had missing service use data. There were no missing cases for demographic and e-PRO data. Median home frequency and mean home involvement summary scores were computed due to non-normal distribution for home frequency scores. Perceived home environmental support summary scores were calculated by summing responses across all home environmental items, dividing the sum by the maximum possible score, and multiplying by 100 (range = 0–100).

We fit a series of multiple linear regression models to estimate children’s functional performance and home participation (frequency and involvement) as a function of EI service intensity, conditional on select child and family characteristics and caregiver perceptions of home environmental support. Variables were selected based on prior models of young children’s participation [16, 17, 20]. Both unadjusted and adjusted regression models were run to examine differences in model fit as a function of the covariates. All regression model estimates were evaluated using confidence intervals. Model fit was assessed via *R*^2^. The values of the residuals were examined to ensure a linear relationship between variables, that the variance of the residuals is constant (homoscedasticity), that the values of the residuals are independent (independent errors), and that the values of the residuals are normally distributed.

## Results

### Characteristics of the study sample

Eighty-four caregivers were approached for study enrollment. Participants were 37 caregivers of children (18 male, 19 female) between 6 and 35 months (SD = 7.7 months). All participants resided in the Denver, Colorado metropolitan area. As shown in Table 1, more than half were caregivers of children 12–23 months (59.5%), White non-Hispanic (82.4%), married (81.1%) with multiple children (59.4%), had a college degree or more (78.3%), were employed (59.9%), and earned greater than $50,000 (73%). Approximately 40% of children were enrolled in center-based childcare.

**Table 1:** Sample characteristics (*N* = 37)

Characteristic	Number (%)
Child sex, male	19 (51.4)
Primary language, English	34 (91.9)
Insurance type, Medicaid and CHP+	7 (18.9)
Had a diagnosis	11 (29.7)
Difficulty accessing respite	10 (27.0)
Child age (months)	
6 to 11	4 (10.8)
12 to 23	22 (59.5)
24 to 35	11 (29.7)
Child race/ethnicity	
White, non-Hispanic	30 (82.4)
Black, non-Hispanic	2 (5.9)
Asian, non-Hispanic	1 (2.9)
Multiracial or other, non-Hispanic	2 (5.9)
Hispanic	2 (5.9)
Child care arrangement**	
Parent or legal guardian	24 (64.9)
Center-based program	9 (24.3)
Family-based program	2 (5.4)
In-home provider	4 (10.8)
Respondent type (mother or female guardian)	34 (91.9)
Employed	22 (59.5)
Education level	
HS or some college	8 (21.6)
College degree	13 (35.1)
Graduate training	16 (43.1)
Family income*	
$0–50,000	9 (24.3)
$50,001–100,000	6 (16.2)
$100,001+	21 (56.8)
Marital status	
Married	30 (81.1)
Single, never married	3 (8.1)
Domestic partner	1 (2.7)
Divorced or separated	3 (8.1)
Siblings	
0	15 (40.5)
1	12 (32.4)
2+	10 (27.0)

*Missing data (*n* = 1)

**Respondents could select multiple responses

As shown in Table 2, children received 7 months of EI on average (SD = 4.8 months), with average EI intensity of 6.1 h per month (SD = 3.3). More than 75% of children received only one EI service. Physical therapy (PT) was the most common EI service (61.8%).

**Table 2:** Early intervention service use characteristics (*n* = 34)

Characteristic	Mean (SD)	
Service duration (months)	7.1 (4.8)	
	*n* (%)	
Type of EI services received*		
PT	21 (61.8)	
OT	9 (26.5)	
SLP	10 (29.4)	
ECSE	4 (11.8)	
Number of EI services received		
1	26 (76.5)	
2	6 (17.7)	
3 or more	2 (5.9)	
	Mean (SD)	Median [IQR]
Total per child EI hours*		
All services	39.5 (28.7)	27.0 [21.3, 53.8]
PT	29.5 (19.6)	26.0 [18.3, 36.3]
OT	28.7 (15.8)	32.0 [13.0, 34.0]
SLP	26.1 (19.3)	26.1 [10.0, 50.5]
ECSE	35.4 (25.9)	32.3 [12.3, 61.6]
EI intensity (hours per month)	6.1 (3.3)	5.9 [3.8, 6.9]

We obtained service use data on 34 out of the 37 children sampled

*EI* early intervention, *PT* physical therapy, *OT* occupational therapy, *SLP* speech-language pathology, *ECSE* early childhood special education

*Small cell count (*n* < 10) for OT and ECSE

### Feasibility of technology-based functional assessment by EI families (aim 1)

Thirty-seven of 84 (44.0%) eligible families enrolled over 2.5 months. Half of 37 subjects enrolled during the EI visit. The remaining 47 eligible families declined due to lack of interest/too busy (19.0%) or privacy concerns (2.4%), were lost to follow up (23.8%), or did not specify (9.5%). Each participant completed both main study questionnaires, as evidenced by no missing e-PRO data.

During the post data collection focus group, EI personnel suggested retaining regular EI-research staff meetings to monitor enrollment, clarify provider questions, and troubleshoot logistical issues. They also recommended retaining multiple options for PRO completion, including iPad during EI visit, personal computer, and phone interview with research staff.

EI personnel proposed three protocol modifications: (1) expand provider training so that more agency staff attend and are provided with a lay summary of study aims and anticipated benefits of e-PROs and summary reports for developing and monitoring EI care plans as part of their routine workflow; (2) focus online data collection during regularly scheduled EI progress evaluations rather than regular service visits to better integrate this type of data collection within routine care; and (3) enable EI personnel to be alerted to summary reports when issued to families, in order to optimize the clinical utility of data gathered.

### Factors associated with parent-reported child functioning (aim 2)

Items on caregiver education and household income were significantly correlated (*r =* 0.65), resulting in the inclusion of education only to decrease multi-collinearity and improve model parsimony. All remaining and significant item associations were less than *r =* 0.49 among pairs of child, family, service, and environmental characteristics; *r* = 0.35 across PEDI-CAT domain scores; and less than *r =* 0.33 between PEDI-CAT and YC-PEM median summary scores. These findings supported inclusion of all study variables separately in aim 2 analyses, results of which are described in the remainder of this section [21].

### Factors associated with children’s functional task performance

PEDI-CAT standard scores for the total sample were within age-expected range for mobility (mean = 47.5, SD = 9.5), daily activities (mean = 52.0, SD = 7.0), and social/cognitive (mean = 52.3*,* SD = 4.5) tasks, with mobility being the lowest and most variable score. As compared to the infants ages 0–6 months, children between 12 and 23 months performed less independently in social/cognitive tasks (*β =* − 4.5, SE = 2.6). There was no significant effect of EI intensity on functional task performance for the sampled children.

### Factors associated with children’s participation in home activities

All sample children, on average, participated in home-based activities about once per week (median *=* 4.38, IQR 4.00, 5.38). More specifically, all children participated once or more each day in basic care routines and interactive and organized play activities, whereas children participated less often in household chores (median *=* 0.00, IQR 0.00, 3.50) and socializing with friends and family (median = 3.50, IQR 2.50, 4.50). Children, on average, were somewhat involved in home activities (mean *=* 3.62, SD = 0.59), and their caregivers wanted their young child’s participation to change in more than half (55.9%) of those activities. Caregivers reported their child’s home environment, on average, somewhat or usually helped with participation in home-based activities (mean = 85.7, SD = 8.5).

There was no significant effect of EI intensity on participation frequency. However, Table 3 shows that caregiver report of their child’s involvement in home activities decreased as a function of greater EI intensity (*β =* − 0.06, SE = 0.02, *p* < 0.05) and less caregiver education (*β =* − 0.47–0.57, *SE* = 0.22–.24, *p* < 0.05) after adjusting for child age, functional performance, and environmental support for participation at home. For every one unit increase in EI service use [EI intensity], caregiver perceptions of the child’s home participation [level of involvement] decreased by 0.06 units. Together, EI service intensity and caregiver education accounted for 51–55% of the variance in home involvement as accounted for through the estimated *R*^2^ for each of the adjusted models.

**Table 3:** Factors associated with parent-reported child involvement in home-based activities

Independent variables	Unadjusted	Adjusted			
		(1)^a^	(2)^b^	(3)^c^	(4)^d^
EI intensity	− 0.03	–	− 0.06*	− 0.06*	−0.06*
	[− 0.09, 0.04]		[−0.10, − 0.01]	[− 0.10, − 0.01]	[−0.11, − 0.01]
Functional performance	0.03**	–	–	0.00	0.00
	[0.01, 0.05]			[− 0.03, 0.03]	[− 0.04, 0.03]
Environmental support	0.00	–	–	–	− 0.01
	[− 0.01, 0.02]				[− 0.03, 0.01]
Child age (months)					
6 to 11	Reference category	Reference category	Reference category	Reference category	Reference category
12 to 23	− 0.33	− 0.27	− 0.36	− 0.36	− 0.42
	[− 1.20, 0.53]	[− 1.01, 0.47]	[− 0.98, 0.26]	[− 1.07, 0.35]	[− 1.12, 0.28]
24 to 35	− 0.01	− 0.04	− 0.12	− 0.12	− 0.17
	[− 0.90, 0.87]	[− 0.77, 0.70]	[− 0.73, 0.49]	[− 0.75, 0.50]	[− 0.79, 0.45]
Caregiver education level					
HS or some college	− 0.49*	− 0.46*	− 0.45	− 0.45	− 0.47
	[− 0.96, − 0.02]	[− 0.91, − 0.01]	[− 0.91, 0.01]	[− 0.92, 0.02]	[− 0.96, 0.02]
College degree	− 0.50*	− 0.46*	− 0.57*	− 0.57*	− 0.57*
	[− 0.93, − 0.08]	[−0.89, − 0.02]	[− 1.01, − 0.13]	[− 1.02, − 0.12]	[− 1.03, − 0.11]
Graduate training	Reference category	Reference category	Reference category	Reference category	Reference category
*R-*squared	–	0.55	0.51	0.52	0.53

Robust confidence intervals in parentheses

** *p* < 0.01, * *p* < 0.05

^a^Adjusted for child and family characteristics (i.e., lowest child age, highest parental education level)

^b^Aadjusted for child and family characteristics listed above in (1), and EI intensity

^c^Adjusted for child and family characteristics listed above in (1), EI intensity, and functional task performance

^d^Adjusted for child and family characteristics listed above in (1), EI intensity, functional task performance, and perceived environmental support in home

## Discussion

This study examined the feasibility and value of implementing technology-based functional assessment into EI practice. This single-site pilot study was not adequately powered to estimate the relationship between EI service intensity and functional status. However, results provide rationale for evaluating these functional outcomes in a scale-up study that will include developmental data from EI records to improve model fit.

Results suggest implementing e-PROs into EI practice is, in general, feasible and contributes clinically relevant knowledge about correlates of children’s participation in home activities. Particularly, prior studies have shown the impact of child, family, and environmental factors on developmental outcomes for young children with developmental disabilities and delays [22], studies of how these characteristics impact young children’s functioning are relatively sparse [18, 23] and do not account for EI service use, which is highly variable [4, 5, 24].

### e-PRO feasibility among EI families

In this study, e-PRO data collection was feasible for nearly half of the EI families sampled when the assessments were introduced to families during EI home visits. Enrollment rates approximated EI family assessment rates, with half of participants engaging in e-PRO completion outside of the EI visit. Data were collected during winter months when there are typically more cancelled or rescheduled appointments, which decreased enrollment due to fewer opportunities for enrolling families and obtaining e-PRO data during EI visits. Alternatively, agency use of a primary service provider model may have decreased enrollment rates because it places high demand on caregiver involvement during EI visits [24]. As a result, providers may have struggled to integrate e-PRO completion during EI visits, resulting in decreased enrollment options for families, particularly caregivers without access to technology for questionnaire completion outside of a routine EI visit.

While e-PRO enrollment rates were slightly lower than usual care, there was a perfect e-PRO completion rate among enrolled families. EI personnel attributed the high completion rate to having flexible options for e-PRO administration. EI staff therefore suggested integrating e-PRO data collection within scheduled family assessments that routinely occur to increase enrollment rates. This change in the timing of e-PRO administration to coincide with a formal evaluation of the child’s functional progress may increase the likelihood of obtaining caregiver input so that EI services can be designed to be responsive to family priorities and functional outcomes can be routinely monitored from the family perspective.

### Modeling home participation outcomes among EI families

In this study, caregiver input about the child’s functioning was obtained in multiple ways through e-PRO completion. While estimates of the child’s functional task performance were within age limits, caregiver concern with the child’s participation was detected through caregiver selection of desiring change in more than half of home-based activities. Caregiver dissatisfaction rates as reported in this study resemble prior studies reporting on young children’s participation difficulty in daycare/preschool [25] and community [10, 11] activities. These results suggest that, like the family interview, e-PRO data can detect caregiver concerns of a child’s functioning to guide family-centered care planning.

Another main premise for using e-PROs in EI is that they may help to build clinically relevant knowledge about the adequacy of EI services for achieving functional outcomes. This study extends prior knowledge about the relative effect of EI service use on functional outcomes [10, 20]. We detected a significant effect of EI intensity on one of three dimensions of the child’s functioning, specifically the child’s level of involvement in home-based activities. Results should be interpreted with caution due to small sample size, but this finding supports the conclusion that e-PRO data on children’s participation may help to accelerate patient-centered outcomes research in EI and therefore merits further study.

The negative association between EI service use and outcomes suggests that children with higher EI service use exhibit greater home participation difficulty. This pilot study is cross-sectional, and the child’s participation was often assessed within the first year of EI service use, whereas rates of change in children’s participation are slow and may take from 1 to 9 years to detect [26] versus months [27]. Therefore, future studies should include repeated e-PRO data collection for 1–3 years if it is feasible to administer as part of routine EI family assessment. These patient-reported outcomes data can then be leveraged to determine EI-specific participation trajectories and links between EI service use and functional outcomes.

We did not find a significant effect of perceived environmental support on young children’s home participation. Prior studies have involved mixed samples of young children with and without developmental disabilities and did not account for the role of EI service use. It is therefore possible that caregiver perceptions of environmental support do not play a significant role in predicting home participation outcomes in the presence of EI services, particularly if those EI services emphasize compensatory intervention approaches that minimize environmental barriers to the child’s participation. We did not have data on EI service type or care quality. Future studies could include additional data extraction from case progress notes and/or administer established parent-reported measures of care quality.

Alternatively, EI intensity was used to capture EI service use in this study. EI intensity provides a more robust measure of EI service use by accounting for both service amount and duration. However, the average duration of EI services among children sampled in this study was only 7 months. Shorter EI duration may have contributed to higher EI intensity rates, thereby resulting in overestimated impact of EI service intensity on functional outcomes. Future studies that time data collection during periodic evaluations of progress will result in a study sample with more variable service duration.

### Limitations

Aim 2 results should be interpreted with caution due to small sample size. Data were collected during the winter season when attendance may be lower, possibly contributing to small sample size. The small sample is predominantly White-identified families with higher education and annual income. One reason for this skewed sample is that YC-PEM is only validated for use with English-speaking families. Provider involvement in data collection was pursued to improve study enrollment. However, provider presence during e-PRO questionnaire completion may have resulted in higher caregiver ratings of their child’s functioning. Finally, parent perspectives on feasibility may inform further protocol modifications prior to scale-up.

## Conclusions

EI has adopted child outcomes reporting to ensure care quality. Family expertise is essential to the task of evaluating the child’s functioning in tasks and activities when in their care. This pilot study provides initial evidence of feasibility and utility in obtaining family input about child functioning for outcomes monitoring. Results suggest that the protocol is feasible with modifications. We have therefore applied these results to inform significant revisions to sampling and methodology for a scale-up study that is underway. This study will include a culturally adapted pilot version of YC-PEM for use with Hispanic families to diversify enrollment and increase generalizability of study findings. In addition, parent satisfaction following e-PRO completion is being obtained to further estimate feasibility.

## Appendix

### Guiding questions

1. Recruitment capability and resulting sample characteristics:

- What is total ENRICH enrollment and total eligible (based on CCTSI inclusion criteria)?
- If we could change one thing about recruitment for the future, what do you suggest we do and why?
- What were the main reasons families didn’t want to participate?

2. Refinement of data collection procedures and outcome measures:

- How did families and/or providers experience data collection?
- What would you suggest we change about provider involvement in data collection?

3. Acceptability and suitability of study procedures:

- How often did participants complete the parts of the study during a service visit?
- Why do you think that some participants didn’t complete the study during a service visit?
- What kind of feedback, if any, did you get from families regarding the reports we sent to them?

4. Resources and ability to manage and implement the study:

- Do you feel that there was enough support from our research team to carry this part of the project out? If we could offer more support, what should that look like?
- Is the technology and equipment sufficient to conduct the study, including collection, management, and analysis of data?

## Abbreviations

COS: Child outcomes summary
ECSE: Early childhood special education
EI: Early intervention
e-PRO: Electronic patient-reported outcome
IDEA: Individuals with Disabilities Education Act
NINDS: National Institute of Neurological Disorders and Stroke
OSEP: Office of Special Education
OT: Occupational therapy
PEDI-CAT: Pediatric Evaluation of Disability Inventory-Computer Adaptive Test
PEM: Participation and Environment Measure
PT: Physical therapy
SLP: Speech-language pathology
YC-PEM: Young Children’s Participation and Environment Measure
